# Protein Phosphatase 2A in the Regulatory Network Underlying Biotic Stress Resistance in Plants

**DOI:** 10.3389/fpls.2016.00812

**Published:** 2016-06-10

**Authors:** Guido Durian, Moona Rahikainen, Sara Alegre, Mikael Brosché, Saijaliisa Kangasjärvi

**Affiliations:** ^1^Department of Biochemistry, Molecular Plant Biology, University of TurkuTurku, Finland; ^2^Division of Plant Biology, Department of Biosciences, Viikki Plant Science Centre, University of HelsinkiHelsinki, Finland

**Keywords:** protein phosphatase 2a, plant immunity, signaling, metabolism, protein phosphorylation

## Abstract

Biotic stress factors pose a major threat to plant health and can significantly deteriorate plant productivity by impairing the physiological functions of the plant. To combat the wide range of pathogens and insect herbivores, plants deploy converging signaling pathways, where counteracting activities of protein kinases and phosphatases form a basic mechanism for determining appropriate defensive measures. Recent studies have identified Protein Phosphatase 2A (PP2A) as a crucial component that controls pathogenesis responses in various plant species. Genetic, proteomic and metabolomic approaches have underscored the versatile nature of PP2A, which contributes to the regulation of receptor signaling, organellar signaling, gene expression, metabolic pathways, and cell death, all of which essentially impact plant immunity. Associated with this, various PP2A subunits mediate post-translational regulation of metabolic enzymes and signaling components. Here we provide an overview of protein kinase/phosphatase functions in plant immunity signaling, and position the multifaceted functions of PP2A in the tightly inter-connected regulatory network that controls the perception, signaling and responding to biotic stress agents in plants.

## Introduction

Modern agricultural practices and breeding programs have significantly increased crop yields over the past century. Compared to their wild ancestors, however, modern crops suffer from reduced stress resistance, since plant breeding has largely focused on increasing yield in monocultural farming. A combination of environmental challenges, such as light stress, drought or plant disease pose major threats for plant health, and the urgent need to control plant disease has recently been exemplified by headlights reporting yield losses caused by the Panama disease of banana or head blight of cereals, caused by the detrimental fungal pathogens *Fusarium oxysporum* and *F. graminearum*, respectively. Besides causing severe symptoms or even death of the plant, biotic and abiotic stresses may deteriorate plant productivity by limiting plant growth and development through impaired physiological functions (Krasensky and Jonak, 2012).

To survive in sub-optimal conditions, plants have evolved a repertoire of mechanisms to combat harmful external cues (Boller and Felix, 2009; Yamazaki and Hayashi, 2015). Recognition of attempted infection or insect infestation leads to reprogramming of basic metabolism and production of deterring chemical compounds to prevent pathogens and pests from colonizing the host plant tissue. Organellar metabolism and signaling are vital in reinforcing these protective reactions, but the exact mechanisms remain poorly understood (Trotta et al., 2014). It is well-known, however, that exposure of plants to excess light promotes formation of protective pigments, such as anthocyanins and other phenolic compounds with distinct antioxidant activities. Such stress-induced plant-derived protective compounds can have both deleterious and beneficial effects in human nutrition, and provide a vast repertoire of molecular structures for discovery of novel bioactive compounds from the plant kingdom. Understanding how the signals arising from recognition of pathogen infection cross-communicate with organelle signaling and become translated into appropriate metabolic adjustments, and how this supports the physiology of the whole cell and disease resistance in the entire plant still requires future research efforts.

Identification of stress-inducible biosynthetic pathways and modeling their integration with the metabolic and regulatory networks governing basic production, stress resistance and growth are increasing trends in modern plant biology (Allahverdiyeva et al., 2015). In these interactions, reversible protein phosphorylation, catalyzed by counteracting activities of protein kinases and protein phosphatases, connects stress perception and the immediate down-stream cascades with the signaling networks that ultimately regulate gene expression profiles and metabolic activities in the cell (Uhrig et al., 2013; van Wijk et al., 2014).

Protein dephosphorylation is emerging as a key regulatory mechanism in plant immunity. In plants, as in other eukaryotic organisms, protein phosphatases can be grouped into four families: PPP (phosphoprotein phosphatase), PPM/PP2C (Mg^2++^- or Mn^2+^-dependent protein phosphatase/protein phosphatase 2C), PTP (phosphotyrosine phosphatase), and aspartate-dependent phosphatase families (reviewed by Uhrig et al., 2013). PPPs and PPM/PP2Cs dephosphorylate serine and threonine residues, and it is believed that the evolutionarily highly conserved PPPs are responsible for catalyzing about 80% of eukaryotic protein dephosphorylation (Moorhead et al., 2009; Lillo et al., 2014). In plants, the PPP family of serine-/threonine phosphatases can be further divided into nine subfamilies: PP1 (protein phosphatase type one), PP2A (protein phosphatase 2A), PP4, PP5, PP6, PP7, SLP (*Shewanella*-like protein) phosphatase, and PPKL (protein phosphatase with Kelch-like repeat domains) (Uhrig et al., 2013). This review highlights the multifaceted function of PP2A in controlling the sensing, signaling and responding to biotic stress agents in plants.

## Sensing and Signaling of Pathogen Infection: Joint Action Among Different Cellular Compartments

### Phosphorelay Signaling from the Plasma Membrane to the Nucleus

**FIGURE 1:**
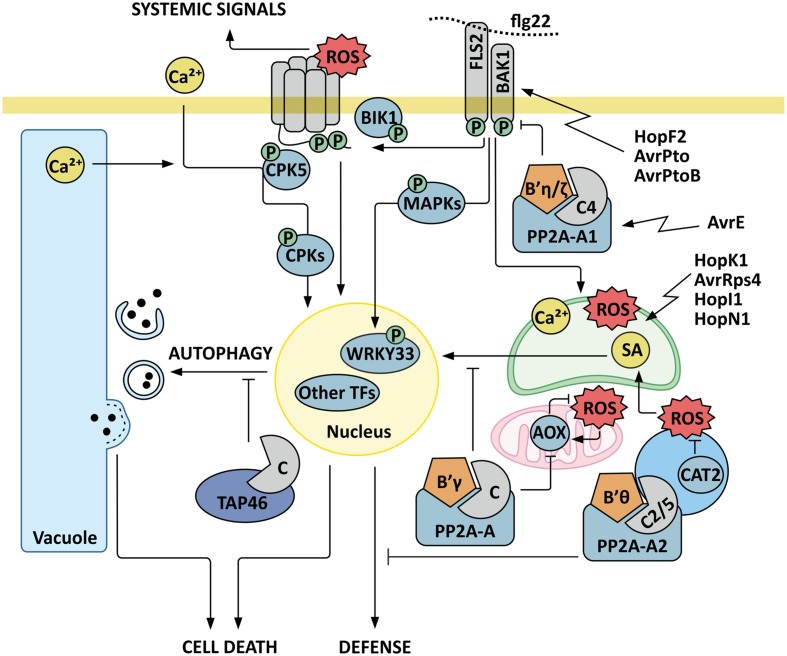
**Model for PP2A as a regulatory component in plant immunity signaling.** Trimeric PP2A protein phosphatase composed of catalytic subunit C4, scaffold subunit A1 and regulatory B subunit B′η or B′ζ negatively regulates the receptor-like kinase BRI1-ASSOCIATED KINASE 1 (BAK1), which is an essential coreceptor of the FLAGELLIN SENSING RECEPTOR 2 (FLS2), and targeted by bacterial effectors HopF2, AvrPto, and AvrPtoB. Activation of FLS2 signaling upon recognition of flagellin (flg22) rapidly elicits transient increases in cytosolic calcium concentration, activation of plasma membrane NADPH oxidases, a consequent burst of reactive oxygen species (ROS) in the apoplast, and activation of MAPKs and calcium-dependent protein kinases (CPKs), which trigger defense gene expression in the nucleus. As an example of a defense-executing phosphorelay cascade, phosphorylation of the transcription factor (TF) WRKY33 by MPKs is depicted. CPK5 additionally mediates systemic ROS signals to distal tissues by activating the NADPH-oxidase RBOHD. Flagellin-induced signals are also rapidly relayed into the chloroplasts, where calcium-dependent signaling interactions trigger chloroplast retrograde signals that further modulate plant immunity. The *P. syringae* effectors HopK1, AvrRps4 HopI1 and HopN1 are indicated as examples that target chloroplastic functions. The PP2A-B′-subfamily members B′𝜃 and B′γ function as negative regulators of plant immunity. PP2A-B′γ controls a feed-back loop where increased abundance of alternative oxidases AOX1A and AOX1D result in reduced ROS production. PP2A-B′γ is also required to control salicylic acid (SA)-dependent pathogenesis responses and cell death triggered by intracellular ROS signals. Another important layer of regulation is provided by the PP2A regulatory protein TAP46, which interacts with PP2A catalytic subunits and negatively regulates autophagy and the associated programmed cell death.

Plants monitor their biotic environment through plasma membrane pattern recognition receptors, which act as receptor-like kinases (RLKs) or receptor-like proteins (RLPs) (Jones and Dangl, 2006; Gust and Felix, 2014) that can sense the presence of conserved pathogen-associated molecular patterns (PAMPs), such as bacterial flagella and peptidoglycans, the fungal cell wall constituent chitin or the saliva of aphids (Gómez-Gómez and Boller, 2000; Liu et al., 2012; Prince et al., 2014; Zipfel, 2014; **Figure 1**). After binding their specific PAMPs, RLKs undergo homo-dimerization or hetero-dimerize with plasma-membrane bound coreceptors, allowing signal initiation by the activated receptor complex. RLPs in turn associate with their corresponding cytoplasmic adaptor kinases either before or after binding their individual pathogen derived ligands to form an active signaling component when the ligand is perceived (Gust and Felix, 2014; Macho and Zipfel, 2014).

Through the cytoplasmic kinase function of RLKs, the recognition rapidly elicits downstream signaling effects that manifest themselves as transient increases in cytosolic calcium concentration, activation of the plasma membrane NADPH oxidases, a consequent burst of reactive oxygen species (ROS) in the apoplast, and concomitant activation of phosphorelay cascades employing mitogen-activated protein kinases (MAPKs) or calcium-dependent protein kinases (CDPKs, or in *Arabidopsis thaliana* CPKs) (Asai et al., 2002; Zhang et al., 2007; Boudsocq et al., 2010; Ranf et al., 2011; Schulz et al., 2013; Savatin et al., 2014). Even though the sequence of events is not yet fully established, these regulatory actions trigger the first line of transcriptional reprogramming in the nucleus. The initial and transient onset of defense gene expression is followed by more persisting changes in hormonal signaling notably through salicylic acid (SA), jasmonic acid (JA), and ethylene (ET), sealing of inter-cellular connections at plasmodesmata, and reprogramming of primary and secondary metabolism (Boller and Felix, 2009; Lee et al., 2011; Chaouch et al., 2012; Wang et al., 2013). To evade PAMP-triggered immunity (PTI), pathogens deliver effector proteins into plant cells to facilitate pathogenesis. As a counter measure, plants have evolved RESISTANCE (R) proteins, which recognize the presence of pathogen effectors and mount a second, stronger response termed effector-triggered immunity (ETI), that is commonly associated with programmed cell death called hypersensitive response (HR) (Jones and Dangl, 2006; Boller and Felix, 2009).

In the context of innate immunity, the MAPKs, which include both positive and negative regulators of PAMP-triggered immunity (PTI), have been well studied (Li et al., 2015). As an example of a defense-executing MPK-phosphorelay cascade, upstream MAPKK-Kinases (MAPKKKs) phosphorylate the MAPK-Kinases (MAPKKs) MKK4 and MKK5, which in turn phosphorylate two closely related MPKs, MPK3 and MPK6 that directly phosphorylate the stress-inducible transcription factor WRKY33. The phosphorylated WRKY33 drives the expression of its immunity-related target genes, such as *1-AMINOCYCLOPROPANE-1-CARBOXYLIC ACID SYNTHASE* (*ACS*) to activate the committing step in ethylene biosynthesis, and *PHYTOALEXIN DEFICIENT 3* (*PAD3*), required to trigger the production of the antimicrobial phytoalexin camalexin (Ishihama and Yoshioka, 2012; Guan et al., 2015). In a recent study, Kang et al. (2015) showed that activated MPK6 additionally phosphorylates the transcription factor BRASSINOSTEROID INSENSITIVE1-ETHYL METHANESULFONATE-SUPPRESSOR1 (BES1) and that this phosphorylation is needed for full resistance against the hemibiotrophic bacterial pathogen *Pseudomonas syringae* pv. *tomato* DC3000 (*Pst*) in *A. thaliana*. Intriguingly, BES1 is a core component of the brassinosteroid signaling pathway, where its brassinosteroid-induced dephosphorylation promotes plant growth through activation of the major gibberellic acid biosynthesis genes (Unterholzner et al., 2015). Together, these signaling interactions illustrate examples of intricate branching among mechanisms that modulate plant growth and defense via key transcriptional regulators.

Parallel to the MPK-mediated signaling mechanisms, transient and presumably highly localized oscillations in cytosolic Ca^2+^ concentration activate a second branch of signal transduction, which is mediated by CDPKs. In *A. thaliana*, concerted action of CPK4, CPK5, CPK6, and CPK11 activates transcription of the immunity-marker genes *NHL10* and *PHI-1* (Boudsocq et al., 2010). CPK5 also initiates systemic signals to distal tissues by mediating Ca^2+^-dependent activation of the NADPH-oxidase RBOHD and a ROS-wave that proceeds in the apoplastic space, leading to the activation of defense responses in non-infected tissues (Dubiella et al., 2013; **Figure 1**). Analysis of calcium signaling interactions in rice, in turn, revealed an intriguing kinase–kinase interaction, where Ca^2+^-activated CPK18 phosphorylates rice MPK5 on threonine 14 an threonine 32, residues that are not part of the general TXY-motif that provides the well-known phosphorylation side for the activation of MAPKs by MAPKKs, and this multi-phosphorylated MPK mediates both activating and repressing effects on its downstream target genes (Xie et al., 2014). Collectively, the delicate regulatory interactions among different types of protein kinases allow subtle changes in gene expression profiles and metabolic pathways, and may therefore specifically adjust the plants’ defense programs against the variety of biotic stress agents.

While protein kinases and their regulation mechanisms have long been intensively studied, the importance of protein phosphatases in plant immunity has been established more recently. The general involvement of protein dephosphorylation in early PTI responses was underscored by quantitative phosphoproteomic analysis of flagellin-treated *A. thaliana* plants, which showed reduced phosphorylation of a number of metabolic enzymes already 15 min after the application of the commonly utilized model for bacterial PAMP, the flagellin epitope flg22 (Rayapuram et al., 2014). In addition to metabolic adjustments, protein phosphatases are evidently also required to limit the activation state of PAMP-triggered phosphorelay cascades. However, even though the transient nature of early defense gene activation (Lewis et al., 2015) speaks for the importance of protein dephosphorylation in limiting the extent of defensive measures, only a few protein kinase–phosphatase pairs with counteracting effects on stress responses in plants have been identified (Schweighofer et al., 2007; Qiu et al., 2008; Geiger et al., 2009; Lee et al., 2009).

An example of a regulatory kinase–phosphatase interaction was provided by Bartels et al. (2009), who showed that the dual-specificity phosphatase MAP KINASE PHOSPHATASE1 (MKP1), together with the PROTEIN TYROSINE PHOSPHATASE1 (PTP1) regulate the phosphorylation status and hence the activity of MPK6. In line with this finding, null mutation of MKP1 resulted in constitutive defense responses and resistance to *P. syringae* (Bartels et al., 2009). Subsequently, Lumbreras et al. (2010) identified MAP KINASE PHOSPHATASE2 (MKP2) as a negative regulator of a programmed cell death, elicited by ectopic overexpression of the *A. thaliana* MPK6 in *Nicotiana tabacum* (Lumbreras et al., 2010). In effector triggered immunity, a MAPK-phosphatase INDOLE-3-BUTYRIC ACID RESPONSE 5 (IBR5) modulates the plant defense response and associated gene expression profiles in response to the bacterial effectors AvrRps4 and AvrRpm1 (Liu et al., 2015). PAMP-induced regulation of gene expression is also controlled through MAPKs that activate cyclin-dependent kinases (CDKCs), which in turn phosphorylate the C-terminal domain of RNA polymerase II, with a consequent activation of defense gene expression Li et al. (2014a). The phosphorylation of these residues is counteracted by dephosphorylation by the protein phosphatase C-terminal domain (CTD) phosphatase-like 3 (CPL3), which functions as a negative regulator of PAMP-induced immune responses (Li et al., 2014a).

Taken together, plant immunity is governed by converging signaling pathways where protein kinases and phosphatases collectively determine the extent of defense reactions. Tight control of immune responses is also critical in preventing unnecessary investment of resources to energy-consuming metabolic changes, such as biosynthesis of secondary metabolites. Connecting the variety of protein kinases to their counteracting protein phosphatases, revealing their target proteins and understanding the physiological significance of the interactions therefore represent outstanding future research questions in plant biology.

### The Centrality of Organelle Biology in Plant Immunity

A few recent studies have provided compelling evidence indicating that chloroplasts, peroxisomes, and mitochondria, with their cross-communicating enzymatic machineries, form active metabolic and regulatory hubs in plant immunity. PAMP-triggered signals are rapidly relayed into the chloroplasts, where calcium-dependent signaling interactions are needed to trigger additional signaling events, which further modulate immunity related gene expression in the nucleus (Nomura et al., 2012; Caplan et al., 2015). It was also recently discovered that stromules, which extend from the chloroplasts to the nucleus during innate immunity, form an important route for defense-associated chloroplast retrograde signaling (Caplan et al., 2015). de Torres Zabala et al. (2015) proposed a scenario where PAMP-triggered signals maintain Photosystem II activity in chloroplasts in order to facilitate a chloroplastic ROS burst and the consequent activation of PTI. On the other hand, suppression of chloroplast-associated genes within the nuclear genome displays another fast defense response in the framework of PTI (Lewis et al., 2015). Since chloroplasts form an essential signaling compartment in plant immunity, pathogens have evolved secreted chloroplast-targeted effector molecules to manipulate the chloroplastic defense mechanisms (Kangasjärvi et al., 2014; Li et al., 2014b; Trotta et al., 2014; de Torres Zabala et al., 2015). For example, the *P. syringa*e effectors HopK1 and AvrRps4 localize to chloroplasts in their processed forms and require chloroplast transit peptides to suppress plant immunity, inferring that their target proteins are present in the chloroplast (Li et al., 2014b). The chloroplast-targeted effectors HopI1 and HopN1 in turn exert their actions by suppressing chloroplastic ROS burst and SA-mediated defenses (Jones et al., 2006; Rodríguez-Herva et al., 2012).

Given that photosynthetic redox signals and oxidative signaling have long been recognized central in triggering abiotic stress responses, notably during light stress (Pogson et al., 2008), it is not surprising that biotic and abiotic stress responses may have partially over-lapping outcomes. Besides the initiating signals arising from the photosynthetic electron transfer chain, a level of cross-talk occurs also within cytosolic stress signaling networks, where light acclimation and biotic stress signaling also appear to share common components. Vogel et al. (2014) reported that MPK6, together with APETALA2/ETHYLENE RESPONSIVE FACTOR- (AP2/ERF-) transcription factors, both of which are commonly associated with plant defense signaling, also mediate light-stress-induced signals that promote rapid and transient light acclimation responses in *A. thaliana*.

Pre-exposure to high light stress has also been shown to enhance plant resistance to subsequent infection by virulent strains of *P. syringae* (Mühlenbock et al., 2008), or infestation by green peach aphid (*Myzus persicae*, Rasool et al., 2014). Even though the concept of such cross-tolerance has become commonly accepted, the developmental and metabolic readjustments that ultimately promote broad-range stress resistance in plants remain to be elaborated (Bowler and Fluhr, 2000; Bostock, 2005). By contrast, the establishment of long term systemic acquired resistance (SAR), which renders uninfected tissue of an infected plant resistant to future infections, is known to involve hydrogen peroxide (H_2_O_2_) and nitric oxide (NO) as well as mono- and digalactosyldiacylglycerol lipids (Gao et al., 2014; Wang et al., 2014). Moreover, the three main regulators of SAR comprise azealic acid, SA, and pipecolic acid (Jung et al., 2009; Bernsdorff et al., 2016), the latter two of which rely on metabolic intermediates derived from the chloroplast.

Besides the generally accepted roles of chloroplasts, peroxisomes and mitochondria in reinforcing plant defenses (Chaouch et al., 2010; Trotta et al., 2014), a signaling role has been realized for foliar lipid bodies, which are small organelles surrounded by a monolayer membrane and which have earlier been regarded as a compartment for lipid storage in seeds. The surface of lipid bodies can accommodate signaling components, such as the pathogen-responsive calcium-dependent protein kinase CPK1 (Coca and San Segundo, 2010). Lipid bodies also store phytosterol esters, which promote plant immunity by modulating the permeability of the plasma membrane, and perform the biosynthesis of the anti-fungal phytoalexin 2-hydroxyoctadecatrienoic acid (2-HOT). Being highly mobile organelles, lipid bodies may also shuttle metabolites and proteins to specific cellular sites of action, hence providing an important addition to the plant’s defense strategies (van der Schoot et al., 2011; Kopischke et al., 2013; Shimada et al., 2014).

Plant immunity is a multilevel process executed through tight communication and metabolic interaction between different cellular compartments, and is highly dependent on the physiological status of the plant. Here we focus on the emerging functions of PP2A in controlling the initiation and transduction of stress signals as well as in modulating organellar signaling and metabolic responses. These actions are vital in ensuring appropriate activation of the plasma membrane sensory systems, downstream signaling cascades and defense gene expression, as well as in the execution of defensive measures through biosynthesis of antimicrobial and insecticidal compounds according to the prevailing environmental cues.

## Protein Phosphatase 2A as a Regulatory Enzyme

Trimeric type 2A protein phosphatases, composed of a catalytic subunit C, scaffold subunit A and regulatory subunit B, are evolutionarily conserved signaling components that essentially regulate stress signaling in animals and plants. The high level of conservation can be exemplified by the *Homo sapiens* PP2A catalytic subunit termed 3FGA_C, which displays approximately 80% amino acid identity with its *A. thaliana* counterparts (Rasool et al., 2014).

Structural analysis of the *H. sapiens* PP2A holoenzyme has shown that the scaffold subunit A forms a horseshoe-shaped fold with its 15 tandem internal repeats of the HEAT (huntingtin-elongation-A subunit-TOR) motif binding the catalytic C subunit (Cho and Xu, 2007). The PP2A-C subunit attains an active conformation upon dimerization with the A subunit, and forms a platform for interaction with the regulatory B subunit. The consensus on PP2A function is that the regulatory B subunit plays the key role in determining specificity of the phosphatase holoenzyme for a substrate (reviewed by Sents et al., 2013). Hence, the regulatory B subunit is commonly referred to as the “specificity unit” that determines the target specificity of the trimeric PP2A holoenzyme (for a review, see Farkas et al., 2007). For a more detailed discussion and comparison of PP2A and its homologs in mammalian and yeast cells we refer the reader to excellent recent reviews by Uhrig et al. (2013) and Lillo et al. (2014).

In plants, each PP2A subunit is encoded by multiple genes (Kerk et al., 2002). The *A. thaliana* genome contains five different genes encoding C subunits, three genes for the A subunits and 17 genes encoding the variable regulatory B subunits, which are further classified as B, B′, and B″ based on their structural characteristics (Farkas et al., 2007; **Table 1**). It is worth noting, however, that the nomenclature for B″-subunits in Farkas et al., (2007) differs from that indicated in the GenBank database^1^ and followed, e.g., by Leivar et al. (2011; **Table 1**). Phylogenetic analysis of plant PP2A catalytic subunits from diverse plant species in turn clustered the C-subunits into two subfamilies designated I and II (He et al., 2004). Within these clusters, the *Arabidopsis* PP2A catalytic subunits C1, C2, and C5 belong to subfamily I while C3 and C4 belong to subfamily II (He et al., 2004). The trimeric holoenzyme compositions provide extensive variability and versatility for PP2A in regulatory networks. What determines the trimeric PP2A subunit composition of a specific holoenzyme is currently not well understood.

**Table 1:** Subcellular localisations of *Arabidopsis thaliana* PP2A regulatory B subunits.

*A. thaliana* PP2A B-subunit	AGI code	Localization	Promoter	Fusion protein	Expression system	Reference
**B- subfamily**					
B alpha (Bα)	AT1G51690	-Cytoplasm -Nucleus -Punctate structures	*UBQ10* AT4G05310	mTurquoise and mVenus	*Nicotiana benthamiana* leaf epidermis	Waadt et al., 2015
B beta (Bβ)	AT1G17720	-Cytoplasm -Nucleus -Punctate structures	*UBQ10*	mTurquoise and mVenus	*N. benthamiana* leaf epidermis	Waadt et al., 2015
**B**′**-subfamily**					
B′ alpha (B′α)	AT5G03470	-Cytoplasm	*pPP2AB*′*α*	YFP	*A. thaliana* (stably transformed)	Tang et al., 2011
		-Cytoplasm -Nucleus	*UBQ10*	mTurquoise and mVenus	*N. benthamiana* leaf epidermis	Waadt et al., 2015
B′ beta (B′β)	AT3G09880	-Cytoplasm	*pPP2AB*′*β*	YFP	*A. thaliana* (stably transformed)	Tang et al., 2011
		-Cytoplasm -Nucleus	*UBQ10*	mTurquoise and mVenus	*N. benthamiana* leaf epidermis	Waadt et al., 2015
B′ gamma (B′γ)	AT4G15415	-Cytoplasm	CaMV *35S*	YFP	*A. thaliana* protoplasts (made from leaves)	Trotta et al., 2011
		-Cytoplasm -Nucleus	CaMV *35S*	YFP	*A. thaliana* protoplasts (made from suspension culture cells)	Matre et al., 2009
		-Cytoplasm -Punctate structures	*UBQ10*	mTurquoise and mVenus	*N. benthamiana* leaf epidermis	Waadt et al., 2015
B′ delta (B′δ)	AT3G26030	-Cytoplasm -Punctate structures	*UBQ10*	mTurquoise and mVenus	*N. benthamiana* leaf epidermis	Waadt et al., 2015
B′epsilon (B′𝜀)	AT3G54930	-Cytoplasm -Nucleus -PM	*UBQ10*	mTurquoise and mVenus	*N. benthamiana* leaf epidermis	Waadt et al., 2015
B′ zeta (B′ζ)	AT3G21650	-Cytoplasm -Mitochondria	CaMV *35S*	YFP (YFP at C-terminus)	*A. thaliana* leaf epidermis (particle bombardment)	Matre et al., 2009
		-Cytoplasm -Nucleus	CaMV *35S*	YFP (YFP at N-terminus)	*N. tabacum* leaf epidermis (particle bombardment)	Matre et al., 2009
		-Cytoplasm -Nucleus -Nucleolus	*UBQ10*	mTurquoise and mVenus	*N. benthamiana* leaf epidermis	Waadt et al., 2015
B′ eta (B′η)	AT3G26020	-Cytoplasm	*pPP2AB*′*η*	YFP	*A. thaliana* (stably transformed)	Tang et al., 2011
		-Cytoplasm -Nucleolus	CaMV *35S*	YFP	*A. thaliana* protoplasts (made from suspension culture cells)	Matre et al., 2009
		-Cytoplasm -Nucleus -Nucleolus -PM -Punctate structures	*UBQ10*	mTurquoise and mVenus	*N. benthamiana* leaf epidermis	Waadt et al., 2015
B′ theta (B′𝜃)	AT1G13460	-Peroxisomes	CaMV *35S*	YFP (YFP at N-terminus)	*A. thaliana* protoplasts (made from suspension culture cells)	Matre et al., 2009
		-Cytoplasm -Nucleus	CaMV *35S*	YFP (YFP at C-terminus)	*A. thaliana* protoplasts (made from suspension culture cells)	Matre et al., 2009
		-Peroxisome-like structures (in leaves and roots)	CaMV *35S*	eYFP	*A. thaliana* (stably transformed)	Kataya et al., 2015b
		-Cytoplasm -Nucleus -Nucleolus -PM	*UBQ10*	mTurquoise and mVenus	*N. benthamiana* leaf epidermis	Waadt et al., 2015)
B′ kappa (B′κ) ^∗^B′iota (B′ι) in GenBank	AT5G25510	-Cytoplasm -Nucleus -Nucleolus -PM -Punctate structures	*UBQ10*	mTurquoise and mVenus	*N. benthamiana* leaf epidermis	Waadt et al., 2015
**B′′-subfamily**						
B″ alpha (B″α)	AT5G44090	-Cytoplasm -Nucleus	*UBQ10*	mTurquoise and mVenus	*N. benthamiana* leaf epidermis	Waadt et al., 2015
B″ epsilon (B″𝜀)	AT5G28850	-Cytoplasm -Nucleus	*UBQ10*	mTurquoise and mVenus	*N. benthamiana* leaf epidermis	Waadt et al., 2015
^∗^B″ beta in GenBank and Leivar et al. (2011)						
B″ delta (B″δ)	AT5G28900	-Cytoplasm -Nucleus	*UBQ10*	mTurquoise and mVenus	*N. benthamiana* leaf epidermis	Waadt et al., 2015
^∗^B″ gamma in GenBank and Leivar et al. (2011)						
B″ gamma (B″γ)	AT1G54450	-Nucleus -Punctate structures	*UBQ10*	mTurquoise and mVenus	*N. benthamiana* leaf epidermis	Waadt et al., 2015
^∗^B″ delta in GenBank and Leivar et al. (2011)						
B″ beta (B″β)	AT1G03960	-Cytoplasm -Nucleus	*UBQ10*	mTurquoise and mVenus	*N. benthamiana* leaf epidermis	Waadt et al., 2015
^∗^B″ epsilon in GenBank and Leivar et al. (2011)						
TON 2 (FASS)	AT5G18580	-Cytoplasm - Mitotic preprophase (root tip cells)	CaMV *35S*	GFP	*A. thaliana* (stably transformed)	Spinner et al., 2013
		-Cytoplasm	CaMV *35S*	GFP	*N. benthamiana* leaf epidermis	Spinner et al., 2013
		-Cytoplasm -Nucleus	*UBQ10*	mTurquoise and mVenus	*N. benthamiana* leaf epidermis	Waadt et al., 2015

*The fusion proteins were examined in transient expression systems unless “stably transformed” is stated. The nomenclature is based on Farkas et al. (2007). For PP2A-B″ subunits and AT5G25510, alternative nomenclature according to either GenBank (http://www.ncbi.nlm.nih.gov/genbank) or GenBank and Leivar et al. (2011) is indicated by asterisks (^∗^).*

Computational analysis indicated that the amino acids located on PP2A-C/A/B interfaces are highly conserved, and hence unlikely to determine the different trimeric compositions of *A. thaliana* PP2A (Rasool et al., 2014). This conclusion is also supported by BiFC experiments in *Nicotiana benthamiana* cells where all three *A. thaliana* scaffold PP2A-A-subunits interacted with all five catalytic PP2A-C-subunits *in vivo* (Waadt et al., 2015). Spatiotemporal gene expression patterns and subcellular localization may instead provide important regulatory levels that determine the availability of the different PP2A subunits for holoenzyme formation (Pernas et al., 2007; Konert et al., 2015a). Indeed, the regulatory PP2A-B subunits have been detected in distinct subcellular localisations, albeit most of them seem to reside in more than one compartment, at least in the over-expression systems currently available for studies on protein localization (**Table 1**).

**FIGURE 2:**
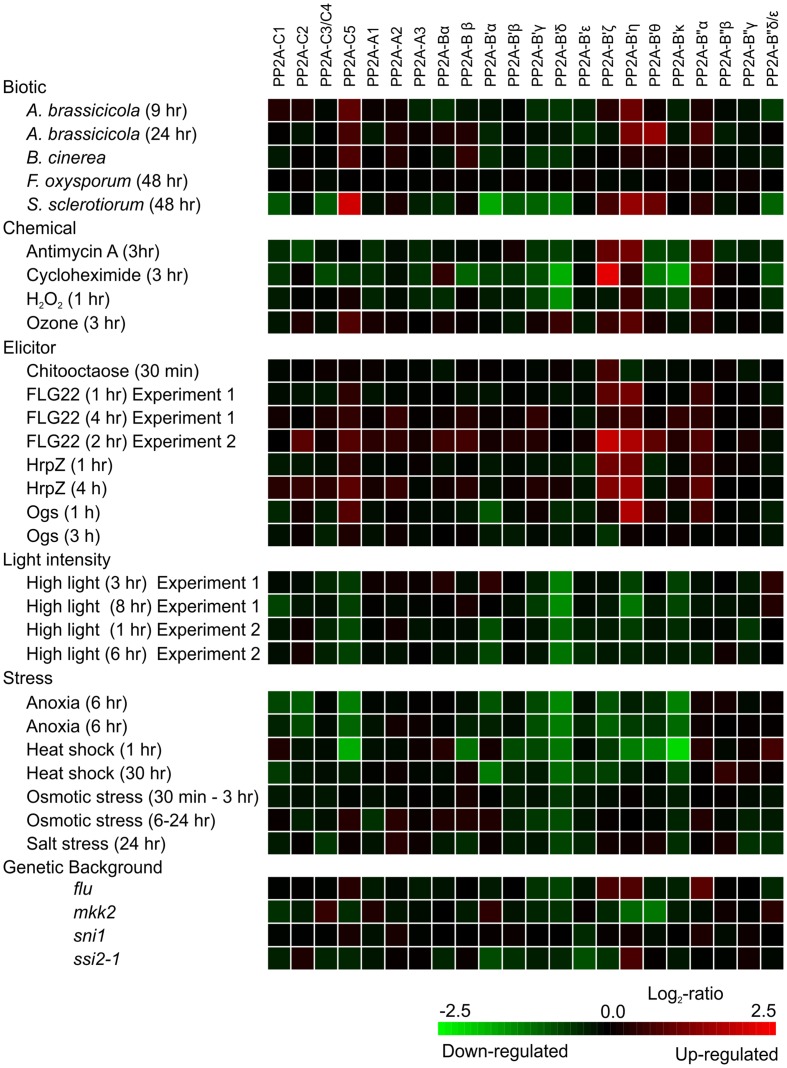
**Expression of PP2A subunits as visualized by investigating selected treatments in Genevestigator using the Perturbations tool.** Green indicates decreased expression and red increased expression. The probes on the Affymetrix ATH1 chip cannot distinguish between *PP2A-C3* and *C4*, *PP2A-B*″δ, and 𝜀 subunits (as named according to Farkas et al., 2007); hence for these genes the indicated expression is the sum of the corresponding genes.

Clues to transcriptional regulation of genes encoding PP2A subunits can be obtained by querying publicly available microarray datasets of mRNA abundance available in Genevestigator^2^ (Hruz et al., 2008) for various experimental conditions (**Figure 2**). Of the catalytic PP2A subunits, *PP2A-C5*, and to some extent also *PP2A-C2* transcript abundance increases in response to various plant pathogens, ozone fumigation that mimics pathogen infection by promoting extracellular ROS burst (Vainonen and Kangasjärvi, 2015) and various pathogenic elicitors, whereas abiotic stresses appear to decrease the expression of the catalytic PP2A subunits. The genes encoding the scaffold A subunits in turn do not seem to be transcriptionally highly responsive. Of the regulatory PP2A-B subunits, the transcript abundance for *B′ζ*, *B′η*, *B′𝜃*, and to some extent also *B*″*α*, follows that of *PP2A-C5*, being increased by biotic cues and reduced by abiotic stress conditions. The transcript abundance for *B*′*α*, *B*′*β*, *B*′γ, and *B*′*δ* in contrast decreases in response to both biotic factors and abiotic stress agents. Hence, transcriptional regulation may provide an important level of regulation of PP2A function.

On the protein level, PP2A activity is modulated through interactions with regulatory proteins, which may be mutually exclusive with PP2A regulatory subunits (Yang et al., 2007; Xing et al., 2008; Wu et al., 2011; Sents et al., 2013). PP2A becomes activated through reversible methylation of a conserved C-terminal leucine residue by the leucine carboxylmethyltransferase (De Baere et al., 1999), which in *A. thaliana* was identified as SUPPRESSOR OF BRI1 (SBI1) in a screen for brassinosteroid signaling components (Wu et al., 2011). This methylation event relies on yet another regulator, the *Arabidopsis* PHOSPHOTYROSYL PHOSPHATASE ACTIVATOR (AtPTPA) (Chen et al., 2014). The elucidation of the *in vivo* function of the PP2A regulator PTPA began originally in yeast and PTPA was shown to be conserved also in mammals (reviewed in Sents et al., 2013). Moreover, both animals and plants also possess other regulatory components, such as the mammalian α4/yeast tap42 (type 2A-phosphatase-associated protein 42 kD) and their homolog TAP46 of *A. thaliana*, which may further modulate the phosphatase activity of PP2A (Harris et al., 1999; Ahn et al., 2011; Sents et al., 2013; Hu et al., 2014). In plants, TAP46 was first described as an interactor of PP2A and PP2A-like phosphatases by Harris et al. (1999). More recent studies have demonstrated key roles for TAP46 in developmental programs and stress responses, but whether and how the TAP46 interaction impacts the catalytic phosphatase activity and/or the target specificity of the protein phosphatase has not yet been indisputably demonstrated (Ahn et al., 2011; Hu et al., 2014; Rexin et al., 2015). Altogether, PP2A is a versatile protein phosphatase that is functionally controlled at multiple levels to ensure specificity in cellular signaling. Furthermore, it is plausible that different nodes of signaling cascades and regulatory networks are controlled by PP2A phosphatases with different heterotrimeric compositions.

## PP2A as a Regulator of Plasma Membrane Sensory Systems

Several recent studies have assigned functions for PP2A in plant immunity. Segonzac et al. (2014) revealed a mechanistic connection between a trimeric PP2A, composed of the catalytic subunit C4, the scaffold subunit A1 and the regulatory B subunits B′η or B′ζ, and the receptor-like kinase BRI1-ASSOCIATED KINASE 1 (BAK1), which is an essential coreceptor of the two extensively studied RLKs FLAGELLIN SENSING RECEPTOR 2 (FLS2) and EF-TU RECEPTOR (EFR) (Segonzac et al., 2014; **Figure 1**). FLS2 and EFR perceive flagellin and the elongation factor (EF-) Tu, respectively, both PAMPs of bacterial pathogens. The PP2A-holoenzyme limits the autophosphorylation and hence the activity of BAK1 (Segonzac et al., 2014). Accordingly, knock-out mutants deficient in the catalytic C4 subunit or the scaffold A1 subunit are hypersensitive to bacterial PAMPs, as demonstrated by increased apoplastic ROS burst upon exposure of leaves to elf18 or flg22, elicitor-active peptides derived from EF-Tu and flagellin, respectively. Associated with this, *pp2a-c4* and *pp2a-a1* show increased resistance to virulent *P. syringae* pv *tomato* DC3000 (Segonzac et al., 2014). These findings are highly significant, given that BAK1 is a key component in various plant-biotic interactions, including the recently discovered resistance to aphids (Prince et al., 2014). The importance of BAK1 as a coreceptor for the key RLKs FLS2 and EFR in the ongoing evolutionary arms-race between plants and their pathogens is also highlighted by the immunity-suppressing effects of the bacterial effectors HopF2, AvrPto and AvrPtoB on BAK1 function (Zhou et al., 2014).

The PAMP-triggered ROS-burst is brought about by the NADPH-oxidase RBOHD, which is activated by the kinases BOTRYTIS-INDUCED KINASE1 (BIK1) and CPK5 (Dubiella et al., 2013; Kadota et al., 2014; Li et al., 2014c), but the protein phosphatases counteracting these phosphorylations for deactivation of RBOHD are yet to be identified. Intriguingly, application of the PP2A-inhibitor cantharidin leads to a ROS-burst with an intensity comparable to that induced by flg22 in *A. thaliana* wild type plants (Segonzac et al., 2014), suggesting that PP2A activity is required to control the NADPH oxidase driven ROS burst. Taken together, PP2A is a key regulatory component that controls PAMP-triggered immunity at receptor level. It is therefore noteworthy that, based on yeast two-hybrid data, Degrave et al. (2015) postulated that a special class of bacterial effectors (AvrEs) may bind to PP2A to promote its function as a negative regulator of plant immunity.

One of the unresolved outstanding questions is how PP2A, which in many cases appears to function as a negative regulator, becomes transiently inactivated upon infection in order to allow elicitation of appropriate defensive or adaptive measures. One possibility is that PP2A becomes a target for a plasma membrane receptor-like kinase, which phosphorylates the attached PP2A phosphatase with a consequent inactivation of the PP2A holoenzyme. Alternatively, pathogen induced ROS signaling could lead to inactivation of PP2A phosphatase activity through oxidative modifications and further nitration or nitrosylation of cysteines of the PP2A proteins. This scenario is supported by a recent report by Van Breusegem et al. (2008), who identified the PP2A regulatory subunit B-α as a target for H_2_O_2_-mediated cysteine oxidation (Waszczak et al., 2014). Such hypothetical regulatory mechanisms, however, call for experimental verification.

## PP2A as a Regulator of Intracellular Signaling Networks and Cell Death

Protein Phosphatase 2A has also been associated with intracellular signaling interactions (**Figure 1**). Apart from the involvement of B′η and B′ζ in containing BAK1-triggered receptor signaling (Segonzac et al., 2014), two other members of the PP2A-B′-subfamily function as negative regulators in plant defense pathways. A mutant deficient in B′𝜃 displays increased resistance toward *P. syringae* pv. *tomato* (Kataya et al., 2015a). B′𝜃 co-localizes with PP2A-C2, PP2A-C5, and PP2A-A2 in peroxisomes, where its positive impact maintains β-oxidation of fatty acids and protoauxins (Kataya et al., 2015b). Whether B′𝜃-dependent regulation of β-oxidation has an impact on plant immunity, however, remains to be established. The subunit B′γ in turn mainly localizes to the cytoplasm where it is required for transcriptional and post-translational control of immune processes, and hence negatively regulates defenses against green peach aphid (*M. persicae*) and the necrotrophic fungus *Botrytis cinerea* (Trotta et al., 2011; Rasool et al., 2014).

Since in *A. thaliana* each regulatory B subunit may theoretically bind 15 different compositions of PP2A, trimeric holoenzymes with different B subunits may regulate cellular functions in both redundant and opposing ways. For example, the regulatory PP2A subunit B′ζ exerts counteracting effects on B′γ-mediated regulation of aphid resistance and cell death in *A. thaliana* (Rasool et al., 2014; Konert et al., 2015a). When compared to wild type, aphid fecundity was decreased in the *pp2a-b*′γ single mutant but not in the *pp2a-b*′γ*ζ* double mutants (Rasool et al., 2014). Acclimation to high light, on the other hand, led to a similar decrease in aphid fecundity in all genotypes (Rasool et al., 2014). These phenotypes are peculiar, given that the B′γ and B′ζ subunits are closely related with 80% amino acid sequence identity (Rasool et al., 2014), albeit their different gene expression profiles speak for differential regulatory roles under biotic stresses (**Figure 2**). Moreover, the saliva-triggered induced resistance against green peach aphid deploys BAK1, which is negatively regulated by B′ζ (Prince et al., 2014; Segonzac et al., 2014). Based on this, B′ζ should rather negatively regulate plant resistance to aphids. Such contradictory phenotypic outcomes presumably reflect the fact that each regulatory PP2A-B subunit is likely to interact with a multitude of target phosphoproteins.

Analysis of plant immunity by using the *pp2a-b*′γ single mutant as a tool is further complicated by its highly conditional phenotype, which depends on light intensity and relative humidity (Trotta et al., 2011; Li et al., 2014d; Rasool et al., 2014; Konert et al., 2015a). When grown under 50% humidity and 130 μmols of photons m^-2^s^-1^ light intensity, *pp2a-b*′γ displays premature yellowing, constitutive accumulation of ROS, SA- and JA- related defense responses, and increased abundance and phosphorylation of pathogenesis related (PR) proteins (Trotta et al., 2011). These phenotypic properties suggest that PP2A-B′γ is required to control both transcriptional and post-translational responses. Growth under 800 μmols of photons m^-2^s^-1^ and elevated temperature, however, partially abolishes the constitutive defense phenotypes and rather promotes enhanced abiotic stress responses and acclimation to high light (Trotta et al., 2011; Konert et al., 2015a; reviewed by Rahikainen et al., 2016). Later studies showed that increasing the growth light from 130 to 200 μmols of photons m^-2^s^-1^ at 65% relative humidity is sufficient to annul the yellowing *pp2a-b*′γ mutant phenotype (Li et al., 2014d). Even though it is evident that PP2A-B′γ is required to control the extent of light acclimation and biotic stress responses in *A. thaliana* leaves (Trotta et al., 2011), these characteristics complicate the interpretation of *pp2a-b*′γ single mutant phenotypes and the understanding of PP2A-regulated processes by mutant approaches.

By taking advantage of pharmacological approaches and selected reaction monitoring (SRM) mass spectrometry, Konert et al. (2015b) showed that *pp2a-b*′γ may partially circumvent oxidative stress through a feed-back loop, where the mitochondrial alternative oxidases AOX1A and AOX1D channel electrons to a bypass pathway with a consequent reduction in ROS production. AOX1A and AOX1D are well known to be transcriptionally responsive to organellar ROS and SA signaling (De Clercq et al., 2013; Sewelam et al., 2014; **Figure 1**). PP2AB′γ, however, exerts its effects on the abundance of AOX1A and AOX1D and mediates post-translational control of the bypass pathway, albeit the molecular mechanism remains unresolved (Konert et al., 2015b). Even so, it is clear that PP2A-B′γ forms a key component within the regulatory network that controls intracellular ROS homeostasis and the involved hormonal signaling responses in *A. thaliana*.

Li et al. (2014d) generated a *catalase 2* (*cat2*) *pp2a-b*′γ double mutant to study the impact of oxidative signaling in the *pp2a-b*′γ mutant background. CATALASE 2 is a major scavenging enzyme that quenches photorespiratory H_2_O_2_ in peroxisomes, and hence provides a highly informative system to study factors involved in intracellular ROS signaling in C3 plants (Mhamdi et al., 2010; Kaurilind et al., 2015). In *cat2*, the oxidative stress induced pathogenesis responses, such as accumulation of SA and phytoalexins, expression of PR genes, lesion formation, and cell death, are conditioned by day length and are more pronounced under long day conditions (Queval et al., 2007, 2012; Chaouch et al., 2010; Li et al., 2014d). Li et al. (2014d) showed that under short photoperiod these ROS-induced pathogenesis responses become suppressed through pathways that require the activity of PP2AB′γ. Physiological and biochemical characterization of *cat2 pp2a-b*′γ double mutants revealed enhanced cell death and reprogramming of primary metabolism, and these adjustments associated with elevated levels of SA and camalexin in short day photoperiods (Li et al., 2014d). Introduction of *sid2* (a mutation in ISOCHORISMATE SYNTHASE1, the major SA biosynthesis enzyme in *A. thaliana*) into the *cat2 pp2a-b*′γ background, however, suppressed the cell death phenotype, demonstrating that the observed pathogenesis responses in *cat2 pp2a-b*′γ were driven by SA signaling (Li et al., 2014d). Hence, PP2A-B′γ is required to control SA-dependent pathogenesis responses triggered by intracellular ROS signals (**Figure 1**).

Besides the regulatory PP2A-B subunits, another important layer of cell death regulation is provided by the PP2A regulatory protein TAP46, which negatively regulates autophagy and the associated programmed cell death, but this pathway appears to be at least partially SA independent (Ahn et al., 2011). In yeast and mammalian cells, Tap42/α4 acts as a downstream component in the target of rapamycin (TOR) protein kinase dependent pathway, which promotes basic cellular functions, such as protein synthesis, transcription and ribosome biogenesis under favorable nutritional conditions. Starvation-induced inactivation of TOR in turn leads to repression of translational activity and recycling of nutrients through the process of autophagy (for a review on TOR signaling, see Jacinto and Hall, 2003; Rexin et al., 2015).

TAP46 was shown to interact with the *A. thaliana* PP2A catalytic subunits, and depletion of TAP46 by virus-induced gene silencing (VIGS) in *N. benthamiana* or RNA interference (RNAi) in *A. thaliana* resulted in global modulations in PP2A activities, albeit the regulatory mode of interaction remains unclear (Ahn et al., 2011). The induced silencing of TAP46 promoted a transient increase in total PP2A activity, which, however, declined in the course of the experiment, leading the authors to conclude that TAP46 may have a more prominent role as an activator of PP2A activity in this experimental system (Ahn et al., 2011). At 7 days of induced silencing, strong depletion of *TAP46* mRNA coincided with reduced PP2A activity and appearance of visually observable cell death on transgenic *A. thaliana* leaves (Ahn et al., 2011). Hence, TAP46 is required to maintain PP2A activity, which in turn prevents unnecessary mounting of the cell death response (**Figure 1**). In the *N. benthamiana* system, depletion of *TAP46* mRNA likewise resulted in growth arrest, activation of autophagy, and programmed cell death, with hallmark features including DNA fragmentation, reduced mitochondrial membrane potential, and decline in chlorophyll fluorescence (Ahn et al., 2011). Hence, depletion of TAP46 phenocopies the physiological effects attributable to inactivation of TOR. Co-expression of the SA-catabolising enzyme NahG delayed the onset of cell death by 4 days, demonstrating that the TAP46-regulated cell death was only partially attributable to SA signaling (Ahn et al., 2011). Rather, the time-dependent modulation of PP2A activity in TAP46-silenced plants coincided with that observed in TOR-deficient plants, and phosphorylation of TAP46 by TOR *in vitro* further inferred that TAP46 may be functionally connected with TOR signaling (Ahn et al., 2011). Even though the exact mechanisms of TAP46/TOR/PP2A interaction remain to be defined, this study highlighted the relevance of PP2A catalytic activity in determining cell death responses in plants.

Catalytic PP2A subunits have also been related to ETI (He et al., 2004), a defense response which commonly includes the HR, a particular type of programmed cell death in plants. By taking advantage of VIGS of subfamily I PP2A catalytic subunits in *N. benthamiana*, He et al. (2004) demonstrated that reduced PP2A activity correlated with increased expression of the pathogens-related genes PR1a, PR1b and PR5 and with localized cell death, which became visually observable in the stems and leaves of the PP2A-silenced plants. Accordingly, the PP2A-silenced *N. benthamiana* plants displayed a 20-fold reduced growth of virulent *P. syringae* pv. *tabaci* as compared to vector-infected control plants, suggesting that the decline in PP2A activity enhances a complex array of mechanisms essential in the impediment of *P. syringae* growth (He et al., 2004).

By taking advantage of *N. benthamiana* transiently expressing the Resistance (R) gene/effector pairs tomato Pto/*P. syringae* pv *tomato* AvrPto or tomato Cf9/*Cladosporium fulvum* Avr9 in PP2A silenced plants, He et al. (2004) could demonstrate that PP2A also negatively controls HR signaling downstream of different R-genes. Furthermore, in response to infection by *P. syringae* pv. *tomato* expressing AvrPto, transcript abundance for the catalytic PP2A subunit *LePP2Ac1* increased in tomato lines capable of recognizing this bacterial effector (Mysore et al., 2002). He et al. (2004) suggested that such transcriptional activation of a negative immune regulator may allow precise regulation of defense pathways to prevent uncontrolled damage to the host tissue. Altogether, PP2A activity is an important contributor to negative regulation of a variety of plant defense responses, notably cell death.

## Executing Plant Immunity: PP2A in the Control of Metabolic Responses

Since biotrophic and hemibiotrophic plant pathogens rely on a supply of metabolites by living plant cells, basic defense mechanisms of the plant employ reprogramming of primary and secondary metabolism as a key mechanism of defense. Such metabolomic restructuring is not only essential in channeling metabolic intermediates for production of deterring secondary compounds, but also in ensuring the supply of energy for the energy-consuming biosynthetic pathways and the withdrawal of nutritional nitrogen-rich compounds from the infection zone. Chaouch et al. (2012) showed that the most highly induced metabolites detected in *A. thaliana* upon infection by virulent or avirulent (i.e., successfully parried by the plant after recognition of a specific pathogen effector) strains of *P. syringae* include the stress hormone SA as well as the monosaccharides ribose and fructose, the amino acids threonine, O-asetylserine, tyrosine, leucine, isoleucine and phenylalanine, and nicotinic acid. These findings reflect the centrality of primary metabolism in plant immunity. In basal defense, metabolic rearrangements are at least partially mediated by PAMP–induced transcriptional activation of genes related to biosynthesis of aromatic amino acids and the down-stream steps for the biosynthesis of secondary metabolites, including anthocyanins, lignins, flavonols, and the phytoalexin camalexin (Truman et al., 2007).

Reprogramming of the plant secondary metabolome with increased amounts of camalexin, agmantine derivatives, and glucosinolates can be triggered by induced expression of constitutively active forms of MPK3 and MPK6 and the ensuing changes in the phosphoproteome, even in the absence of pathogen infection (Lassowskat et al., 2014). This finding highlights protein phosphorylation as the key mechanism in triggering inducible chemical defenses in plants. Cruciferous plants harbor a particularly complex set of secondary compounds, of which camalexin and indole glucosinolates have well-documented antimicrobial effects in different *Arabidopsis* accessions, which have been instrumental in the identification of the underlying biosynthetic pathways (Sønderby et al., 2010). Post-translational regulation and its impact on defense-associated metabolism in contrast remains less well understood.

### PP2A in the Regulation of Primary Metabolism

Proteomic and metabolomic analysis of *pp2a-b*′γ and *cat2 pp2a-b*′γ mutants provided insights into potential PP2A targets in post-translational regulation of immune reactions (Trotta et al., 2011; Li et al., 2014d). Besides the recognized key players in pathogenesis responses, such as the pathogenesis-related proteins PR2 and PR5, these studies revealed enzymes of primary metabolism as potential PP2A targets, and suggested that PP2A-B′γ influences the post-translational regulation of oxidative-stress-triggered modulations in primary and secondary metabolism.

**FIGURE 3:**
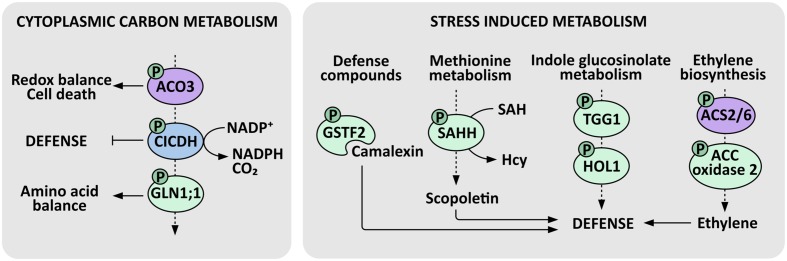
**PP2A-regulated enzymes in defense metabolism.** Cytosolic aconitase (ACO), isocitrate dehydrogenase (CICDH) and glutamine synthase form a metabolic cascade that is tightly connected with defensive responses in plants. PP2A-B′γ regulates the phosphorylation of cytosolic ACONITASE 3, which contributes to redox balance and cell death regulation. ICDH prevents over-amplification of defense responses and its activity yields α-ketoglutarate, CO_2_, and NADPH. Further down-stream, PP2A-B′γ exerts a control over mRNA and protein abundance of a defense-associated GLUTAMINE SYNTHASE 1;1 (GLN1;1), which contributes to remobilization of amino acids by assimilating α-ketoglutarate and ammonia, and may have a key role in the maintenance of redox balance in chloroplasts. Primary metabolism is also tightly connected with stress-induced metabolism. PP2A-B′γ controls the mRNA and protein abundance of the camalexin binding protein GSTF2, which is a phosphoprotein possibly involved in the transport of this deterring defense compound. PP2A-B′γ also controls the key activated methyl cycle enzyme *S*-adenosyl-homocysteine hydrolase (SAHH), which contributes to the recycling of *S*-adenosylmethionine (SAM), hence maintaining transmethylation reactions essential in the biosynthesis of a vast range of protective compounds, such as scopoletin and methylated indole glucosinolates. PP2A-B′γ is also required to control the accumulation and phosphorylation of the myrosinase TGG1 and the thiocyanate methyltransferase 1 (HOL1), which methylates the thiocyanate arising from the degradation of indole glucosinolates. SAM also serves as a precursor for the biosynthesis of the stress hormone ethylene, where the ACC synthases ACS2 and ACS6 and possibly also ACC oxidase 2 are controlled by PP2A. Demonstrated PP2A targets are indicated by violet, and enzymes with increased protein amount and/or phosphorylation in a *pp2a-b*′γ mutant background are indicated by green. ICDH has been identified as a phosphoprotein in the *Arabidopsis* Protein Phosphorylation Site Database (http://phosphat.uni-hohenheim.de/) but its regulation has not been connected with PP2A and is hence indicated in blue. P denotes phosphoprotein.

Attempts to identify the molecular mechanisms underlying PP2A dependent metabolic adjustments revealed that PP2A-B′γ interacts with and regulates the phosphorylation level of the cytosolic form of ACONITASE 3 (Konert et al., 2015b; **Figure 3**). The *A. thaliana* genome encodes three aconitase isoforms that are all dually localized to mitochondria and cytosol. Like ACONITASE 1 and ACONITASE 2, ACONITASE 3 also operates in the mitochondrial citric acid cycle, but it is additionally the key isoform that executes cytoplasmic functions and essentially contributes to energy metabolism at least in young *A. thaliana* seedlings (Hooks et al., 2014). Cytosolic aconitases act in a metabolic cascade where the aconitase-driven reaction is followed by successive activities of isocitrate dehydrogenase (ICDH) and glutamine synthase, both of which have been functionally connected with plant immunity (Mhamdi et al., 2010; Seifi et al., 2013). Intriguingly, PP2A-B′γ exerts a control over transcript an protein abundance of cytosolic glutamine synthase 1;1 (GLN1;1), which functions in remobilization of nitrogen-rich amino acids and may have a key role in maintaining chloroplast redox balance in infected leaves (Liu et al., 2010; Trotta et al., 2011; **Figure 3**). Even though the exact mechanisms remain to be reported, PP2A and primary carbon metabolism appear to be tightly intertwined in triggering defensive measures in plants.

Besides the role in primary carbon metabolism, aconitases also contribute to the regulation of cellular redox balance and cell death in plants (Moeder et al., 2007). *A. thaliana aconitase 3* mutants and *N. benthamiana* plants with reduced aconitase levels displayed increased resistance against methyl viologen induced oxidative stress (Moeder et al., 2007). This effect may be at least partially connected to the fact that aconitases possess iron in their active center, and this iron may be readily released upon attack of the iron–sulfur cluster by ROS. Such ROS-induced release of iron forms a potential for an even more drastic ROS burst through formation of hydroxyl radicals in the Fenton reaction (Navarre et al., 2000). This provides a plausible explanation for the observed oxidative stress tolerance of the aconitase-deficient plants, and raises a question whether PP2A-mediated dephosphorylation of ACONITASE 3 contributes to cellular ROS homeostasis by modulating the stability of the iron–sulfur cluster.

### The Impact of PP2A on Stress-induced Secondary Metabolism and Hormonal Signaling

The *cat2 pp2a-b*′γ double mutant proved instrumental in the analysis of PP2A-dependent regulation of secondary metabolism (Li et al., 2014d). Metabolite profiling and subsequent hierarchical clustering of the quantified metabolites revealed only minor impacts caused by the *pp2a-b*′γ and *cat2* single mutations on the global metabolite contents when examined in short-day conditions, which are non-permissive for induction of pathogenesis responses in *cat2* (Li et al., 2014d). The *cat2 pp2a-b*′γ double mutant in contrast showed a striking metabolic signature, which significantly over-lapped with those obtained in long day grown *cat2* or the *P. syringae* challenged wild type leaves (Chaouch et al., 2010, 2012). This signature included marked changes in the contents of amino acids and their derivatives, exemplified by tryptophan, SA and camalexin (Li et al., 2014d). As discussed above, camalexin biosynthesis becomes transcriptionally activated in PTI upon infection of *A. thaliana* plants by a large variety of microorganisms, including bacteria, fungi, and oomycetes, and is commonly recognized as an outcome of SA and ROS signaling (Glawischnig, 2007; Van Breusegem et al., 2008). However, perturbations in the function of PP2A-B′γ did not significantly alter the expression of camalexin-related genes, with the exception of the gene encoding the camalexin binding protein GSTF2 (Trotta et al., 2011; Li et al., 2014d), which also displayed increased abundance and phosphorylation in *pp2a-b*′γ (Li et al., 2014d). This further associated with a slight increase in camalexin content in the constitutively defense-active *pp2a-b*′γ single mutant and a significant accumulation in *cat2 pp2a-b*′γ (Li et al., 2014d; **Figure 3**). Thus, PP2A-B′γ appears to be required for post-translational control of oxidative-signal-induced metabolic alterations in *A. thaliana* leaves.

2D-gel electrophoresis and subsequent mass spectrometry analysis identified a number of proteins the abundance and/or phosphorylation of which was altered in *cat2 pp2a-b*′γ (Li et al., 2014d: **Figure 3**). These proteins included the central activated methyl cycle enzyme *S*-adenosyl-homocysteine hydrolase (SAHH), which contributes to the recycling of methionine, a metabolic function essential in plant resistance to pathogens. Stress-induced biosynthetic processes involve a vast range of transmethylation reactions, which form a significant sink for the methyl donor *S*-adenosylmethionine (SAM). The reaction product *S*-adenosyl-homocysteine (SAH) is toxic and must be continuously hydrolyzed by SAHH, making this an essential step in the recycling of methionine and SAM and the associated production of a wide range of defense-related secondary metabolites (Moffatt and Weretilnyk, 2001). In *cat2 pp2a-b*′γ, SAM is consumed in the biosynthesis of scopoletin, a coumarin that accumulates in response to biotic stress agents (Chong et al., 2002; Li et al., 2014d). On protein level, *cat2 pp2a-b*′γ mutants exhibit increased accumulation and phosphorylation of the myrosinase TGG1 involved in the catabolism of indole glucosinolates, and the thiocyanate methyltransferase 1 (HOL1), which in turn methylates the thiocyanate arising from the *in vivo* degradation of indole glucosinolates (Nagatoshi and Nakamura, 2009), suggesting that indole glucosinolate metabolism is under the control of PP2A-B′γ (Li et al., 2014d; **Figure 3**).

Yet another sink for SAM is the biosynthesis of ethylene, which plays a role in plant resistance against bacterial and fungal pathogens (Guan et al., 2015). Ethylene biosynthesis is highly induced in both PTI and ETI, and a recent report by Guan et al. (2015) showed that this induction is potentiated by SA signaling through a pathway that largely depends on MPK3 and MPK6 and their downstream targets, the ACC synthases ACS2 and ACS6. Using the *roots curl in naphthylphthalamic acid 1* (*rcn1*) mutant as a tool, Alison DeLong and co-workers showed that the same ACS2 and ACS6 isoforms are negatively controlled by PP2A-dependent dephosphorylation (Skottke et al., 2011). As a consequence, *rcn1* mutants display constitutive ethylene production, which was, however, not further enhanced in response to flagellin-induced signals (Skottke et al., 2011). Li et al. (2014d) found that also ACC oxidase 2, which mediates the next enzymatic step in the biosynthesis of ethylene, is a phosphoprotein with significantly elevated protein level in *cat2 pp2a-b*′γ double mutants. Hence, PP2A may, directly or indirectly, negatively regulate multiple enzymatic steps in stress-induced ethylene biosynthesis.

In conclusion, the multitude of PP2A-dependent metabolic adjustments reflect the intimate connections between primary and secondary metabolism and cellular signaling. These interactions can be readily adjusted by protein kinases and protein phosphatases that delicately fine-tune different levels of plant immunity.

## Author Contributions

All authors listed, have made substantial, direct and intellectual contribution to the work, and approved it for publication.

## Conflict of Interest Statement

The authors declare that the research was conducted in the absence of any commercial or financial relationships that could be construed as a potential conflict of interest.

## Footnotes

1 http://www.ncbi.nlm.nih.gov/genbank

2 www.genevestigator.com
